# HERV-W and *Mycobacterium avium* subspecies *paratuberculosis* Are at Play in Pediatric Patients at Onset of Type 1 Diabetes

**DOI:** 10.3390/pathogens10091135

**Published:** 2021-09-03

**Authors:** Marta Noli, Gianfranco Meloni, Pietro Manca, Davide Cossu, Mario Palermo, Leonardo A. Sechi

**Affiliations:** 1 Dipartimento di Scienze Biomediche, Università degli Studi di Sassari, 07100 Sassari, Italy; martanoli@outlook.it (M.N.); Dcossu@uniss.it (D.C.); 2 Dipartimento di Medicina Mediche, Chirurgiche e Sperimentali, Università degli Studi di Sassari, 07100 Sassari, Italy; gfmeloni@uniss.it; 3 Servizio Centro Trasfusionale, Azienda Ospedaliera Universitaria Sassari, 07100 Sassari, Italy; pietro.manca@aousassari.it; 4 Servizio di Endocrinologia, Azienda Ospedaliera Universitaria Sassari, 07100 Sassari, Italy; mario.palermo@aousassari.it; 5 Struttura Complessa di Microbiologia e Virologia, Azienda Ospedaliera Universitaria Sassari, 07100 Sassari, Italy; 6 Mediterranean Center for Disease Control, Università degli Studi di Sassari, 07100 Sassari, Italy

**Keywords:** HERV-W, *Mycobacterium paratuberculosis*, antibodies, molecular mimicry, proinsulin, children T1D onset, peptides

## Abstract

The etiology of T1D remains unknown, although a variety of etiological agents have been proposed as potential candidates to trigger autoimmunity in susceptible individuals. Emerging evidence has indicated that endogenous human retrovirus (HERV) may play a role in the disease etiopathogenesis; although several epigenetic mechanisms keep most HERVs silenced, environmental stimuli such as infections may contribute to the transcriptional reactivation of HERV-Wand thus promote pathological conditions. Previous studies have indicated that also *Mycobacterium avium* subspecies *paratuberculosis* (MAP) could be a potential risk factor for T1D, particularly in the Sardinian population. In the present study, the humoral response against HERV-W envelope and MAP-derived peptides was analyzed to investigate their potential role in T1D etiopathogenesis, in a Sardinian population at T1D onset (*n* = 26), T1D (45) and an age-matched healthy population (*n* = 45). For the first time, a high serum-prevalence of anti-Map and anti-HERV-W Abs was observed in pediatric patients at onset of T1D compared to T1D patients and healthy controls. Our results support the hypothesis that external infections and internal reactivations are involved in the etiology of T1D, and that HERV-W activation may be induced by infectious agents such as MAP.

## 1. Introduction

Type 1 diabetes (T1D) is an autoimmune disorder afflicting millions of people around the world, characterized by T cell infiltration within pancreatic islets and marked by the production of autoantibodies to the beta-cell [1]. The destruction of 90% of beta cells is related to the onset of the clinical symptoms [2]. The major genetic determinant of T1D are polymorphisms within the HLA region [3], although over 60 non-HLA loci T1D susceptibility loci have been identified [4]. However, the genetic drift alone cannot explain the increase in the incidence of T1D, and environmental factors are thought to play a critical role in triggering islet autoimmunity [5,6]. A variety of studies suggested that the environmental agents trigger disease development in genetically susceptible subjects [7,8]. The loss of self-tolerance and the development of the immune response can be a consequence of the mechanism of cross-reactivity associated with the molecular mimicry provoked by infectious factor [9]. A number of factors, including diet, intestinal dysbiosis, viral infections such as enteroviruses (coxsackieviruses, rotaviruses, cytomegalovirus, parvovirus), have been explored as potential risk factor for the disease progression, but no causal relationship has been established [8].

Previous studies have indicated that *Mycobacterium avium* subspecies *paratuberculosis* (MAP) could be a potential risk factor for T1D [10,11]. MAP is the etiological agent of Johne’s disease, a chronic and inflammatory bowel disease that affects ruminants [12]. The primary route of MAP transmission is oral-fecal through ingestion of contaminated milk, water or the environment caused by infected animals [13,14,15].

MAP is able to invade the intestinal mucosa by interacting with enterocytes [16]. MAP is endemic in the ruminant population of Sardinian Island, an area with one of the highest incidences of T1D in the world [17] and numerous clinical studies have linked MAP to T1D in the Sardinian population [10]. We previously identified different MAP peptides based on the sequence homologies present with human proteins including zinc transporter protein 8 (ZnT8) and with proinsulin (PI) and use them in ELISA [10].

Emerging evidence has also indicated that human endogenous retrovirus (HERV) may play a role in T1D etiopathogenesis [18,19], and it has been reported to function also as a superantigen due to the ability to induce a strong T cell responses [20].

Part of the human genome, about 8%, is composed of HERV, which until recently were considered junk DNA. The integrations of the provirus genome into the DNA of germinal cells are the remnants of infections that occurred over several million years; however, numerous nonsense mutations have rendered them defective [21]. The envelope proteins of HERV-W (HERV-Wenv) has been detected in serum, in peripheral blood mononuclear cells and within the pancreas of patients with T1D [18,19,20], also, in an in vitro study in transgenic mice, HERV-Wenv repressed internal insulin secretion [22,23]. The monoclonal antibody GNbAC1, has been developed to neutralize the effects of HERV-Wenv, and is currently in phase IIa clinical trials as a possible HERV-based therapeutic approach in T1D [24]. Interestingly, not all HERVs remain silenced, and they can be reactivated under certain pathological condition such as infection [24]. The aim of this study was to investigate the role of MAP and HERV in a Sardinian pediatric population with T1D, analyzing the humoral response against homologues peptides derived from both pathogens.

Collectively, data obtained from this study support the involvement of MAP and to HERV-Wenv in the loss of immune tolerance that leads to autoimmunity in T1D. Furthermore, a positive correlation between anti-MAP and anti- HERV-Wenv antibodies observed, suggested a potential relationship between MAP seropositivity and reactivation of HERV.

## 2. Materials and Methods

### 2.1. Patients 

A total of 45 children with T1D pediatric and young patients (median age 12.6, *n* = 18 females and *n* = 27 males mean years with T1D = 6) and 26 patients with onset of T1D (median age 7.8, *n* = 7 females and *n* = 19 males) recruited at the time of diagnosis and blood sampling was performed before starting with insulin therapy, were enrolled in this retrospective study. The inclusion criteria were diagnosis of T1D upon manifestation of the clinical symptoms such as severe hyperglycemia e/o ketoacidosis, testing for the presence of classical islet autoantibodies, as well as levels of glycated hemoglobin, according to the American Diabetes Association criteria [25]. Clinical data of T1D patients are available in Supplementary Table S1. Forty-five healthy and age-matched (median age 11.8 years, 29 females, 16 males) volunteers without autoimmune diseases and inflammatory episodes in the last 2 months served as controls. Patients were recruited at the Pediatrics department of the AOU of Sassari and Department of Experimental and Clinical Medicine of the University of Sassari, while controls samples were obtained from the Department of Endocrinology of the University Hospital (AOU) of Sassari and written informed consent from a parent or legal tutor was obtained for all study participants. All methods were performed in accordance with regional and national regulations.

### 2.2. Blood Samples

Five to eight mL of peripheral blood was taken from each individual in EDTA tubes which was processed within 12 h of collection. Plasma was obtained using Ficoll-Paque^®^ (Cynthia Europe Milano, Italy) according to the protocol. Plasma rates was immediately transferred into a clean polypropylene tube and apportioned into 0.5 mL aliquots and transported at –20 °C for short-term storage (<6 months) and −80 °C for long-term storage (>6 months). 

### 2.3. Peptides 

Peptides showed in Table 1: MAP 3865c _125–133_ (MIAVALAGL), MAP 3865c _133–141_ (LAANFVVAL), MAP 2404c _70–85_ (RGFVVLPVTRRDVTDV) and MAP 1,4-α-gbp _157–173_ (GTVELLGGPLAHPFQPL), HERV-Wenv _93–108_ (NPSCPGGLGVTVCWTY), HERV-Wenv _129–143_ (VKEVISQLTRVRHGT), HERV-Wenv _248–262_ (NSQCIRWVTPPTQIV) were synthesized at >95% purity (LifeTein, South Plainfield, NJ, USA) assessed by HPLC. The peptides were resuspended in 10 mM of dimethyl sulfoxide (DMSO) and kept in single-use aliquots at −80 °C.

### 2.4. Enzyme-Linked Immunosorbent Assay (ELISA) 

Ninety-six-well plates (Nunc-Immuno™ Plates, Maxi Sorp, Nalgen Nunc International, Rochester, NY, USA) were coated overnight at 4 °C with 10 µg/mL of each peptide in a solution 0.05 M of carbonate-bicarbonate, pH 9.5 (Sigma-Aldrich, St. Louis, MO, USA). Plates were washed two times with 0.1% Tween-20 (in TBS) and blocked with 5% skimmed dried milk in PBS for 1 h at room temperature (25 °C). Plasma samples (1:1000 dilution) were added and incubated for 2 h at room temperature. Secondary antibody was alkaline phosphatase-conjugated goat anti-human immunoglobulin G polyclonal Ab (1∶1000; Merk Life Science S.r.l., Milano, Italy). Plates were washed between each incubation. Alkaline phosphatase was detected with para nitrophenyl phosphate (Merk Life Science S.r.l., Milano, Italy). Absorbance was read at 405 nm on a plate reader (SpectraMax Plus 384, Molecular Devices, Sunnyvale, CA, USA). All incubation volumes were 100 μL/well.

Each sample was run in duplicated, and normalization was performed with a positive control (absorbance reactivity set at 1.0 arbitrary units) included in each assay. Background activity was calculated as the mean signal of an immobilized peptide with secondary Ab alone.

### 2.5. Statistical Analysis

GraphPad Prism 9.0 software (GraphPad Software, San Diego, CA, USA) was used for calculating the sensitivity and specificity, false negative and false positive values. Mann–Whitney test were used to analyze non-parametric data. For evaluation of diagnostic values of Ab, receiver operating characteristic curve (ROC) and area under the curve (AUC) was established. Fisher’s exact test and Spearman’s were used for comparison and correlation between MAP and HERV-Wenv peptides, respectively; *p* < 0.05 was considered statistically significant.

## 3. Results

### 3.1. Seroreactivity against MAP Antigens

**Figure 1:**
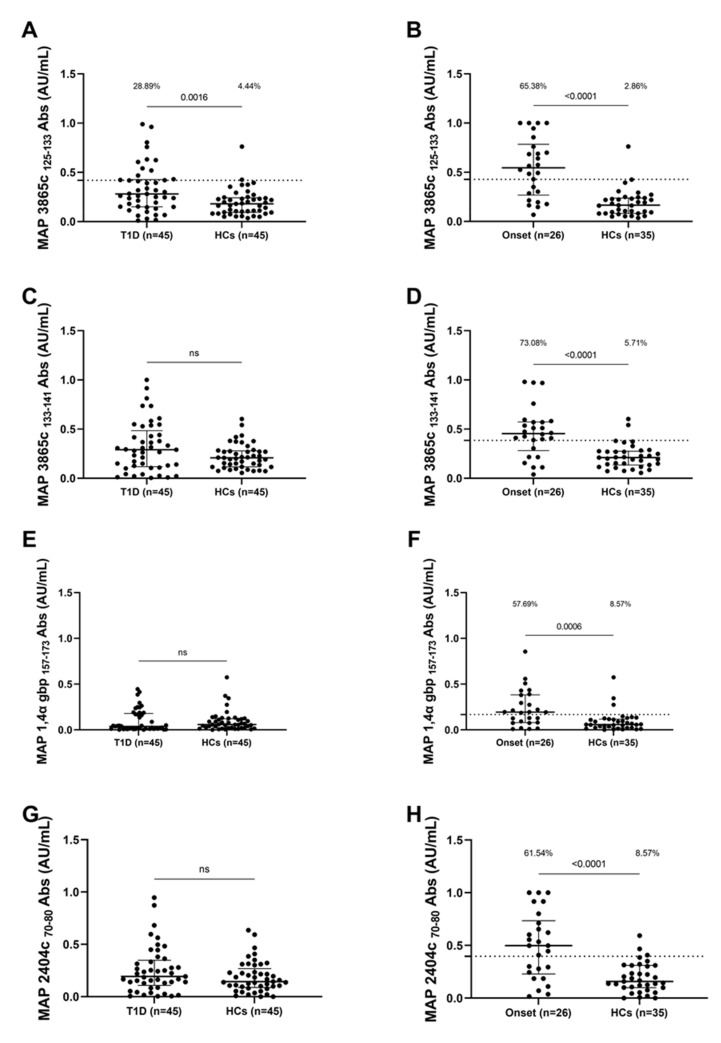
Prevalence of Abs against MAP antigens in Sardinian T1D children. Plasma samples from T1D patients, HCs and patients at T1D onset were tested against MAP 3865c _125–133_ (**A**,**B**), MAP3865c _133–141_ (**C**,**D**), MAP 1,4-α-gbp _157–173_ (**E**,**F**) and MAP 2404c _70–80_ (**G**,**H**) peptides. The dotted lines represent positivity thresholds calculated by ROC analysis; Mann–Whitney *p*-value and the percentage of positive patients evaluated by Fisher’s exact test are indicated in the upper part of each graph.

Significant differences were detected between the patient’s groups and the control group in terms of positivity and mean levels of anti-MAP Abs. The positivity and mean levels of anti-MAP 3865c _125–133_ Abs were significantly more frequent in T1D patients (28.89%, 13 out of 45) than in the healthy controls (HCs) (4.44%, 2 out of 45) (cut-off value of 0.41; AUC = 0.69; *p* < 0.0016, Figure 1A), and were significantly higher in patients with onset of T1D (65.38%, 18 out of 26) than in HCs (2.86%, 1 out of 35) (cut-off value 0.42; AUC = 0.84; *p* < 0.0001, Figure 1B). Anti-MAP 3865c _133–141_ Abs were detected in patients with onset of T1D (73.08%, 19 out of 26 ) and in HCs (5.71%, 2 out of 35) (cut-off value 0.38; AUC = 0.80; *p* < 0.0001, Figure 1D); anti-MAP 1,4-α-gbp _157–173_ Abs were detected in patients with onset of T1D (57.69%, 15 out of 26 ) and HCs (8.57%, 3 out of 35) (cut-off value 0.16; AUC = 0.75; *p* = 0.006, Figure 1F); anti-MAP 2404c _70–80_ were detected in patients with onset of T1D (61.54%, 16 out of 26) and HCs (8.57%, 3 out of 35) (cut-off value 0.39; AUC = 0.78; *p* < 0.0001, Figure 1H). No significant differences were detected between T1D patients and HCs concerning the antibody response against MAP 3865c _133–141_ (Figure 1C), MAP 1,4-α-gbp _157–173_ (Figure 1E), MAP 2404c _70–80_ (Figure 1G).

### 3.2. Seroreactivity against HERV-W Antigen

**Figure 2:**
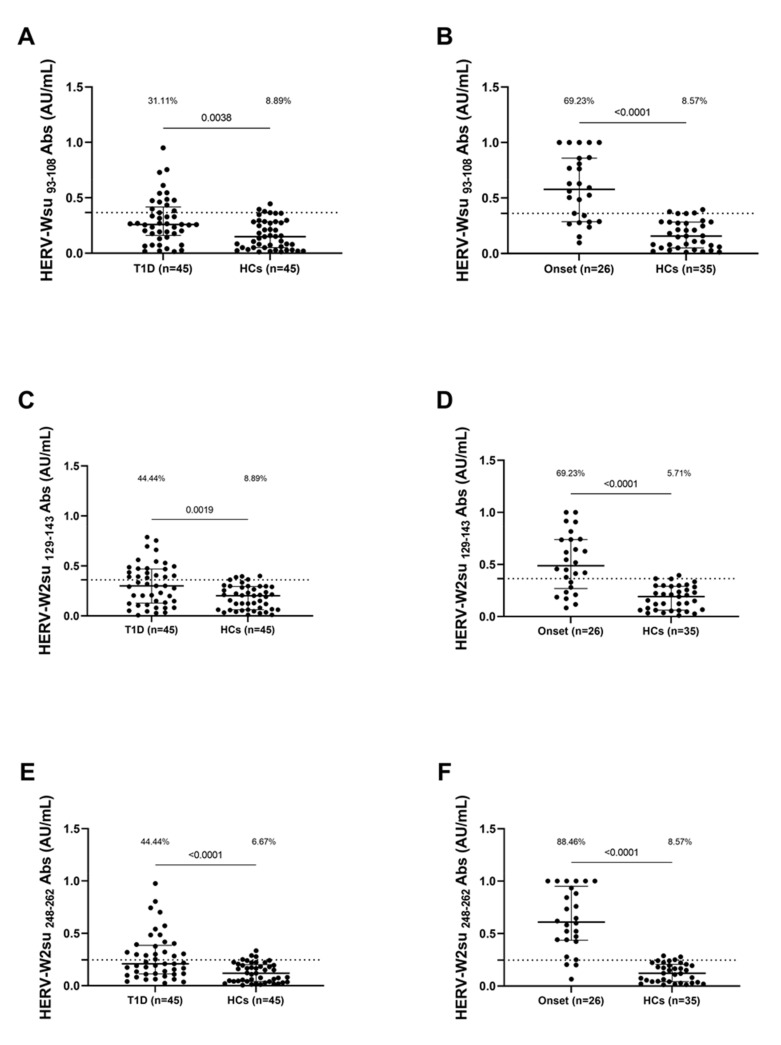
Prevalence of Abs against HERV-Wenv antigens in Sardinian T1D children. Plasma samples from T1D patients, HCs and patients at onset were tested against HERV-Wenv _93–108_ (**A**,**B**), HERV-Wenv _129–144_ (**C**,**D**) and HERV-Wenv _248–262_ (**E**,**F**) peptides. The dotted lines represent positivity thresholds calculated by ROC analysis; Mann–Whitney *p*-value and the percentage of positive patients evaluated by Fisher’s exact test are indicated in the upper part of each graph.

Concerning HERV-W peptides, significant differences in Ab distribution were also detected between the patient groups and the control group. Anti-HERV-Wenv _93–108_ Abs were detected in T1D patients (31.11%, 14 out of 45) and in HCs (8.89%, 4 out of 45) (cut-off value of 0.36; AUC = 0.67; *p*  =  0.0038, Figure 2A); in patients with onset of T1D (69.23%, 18 out of 26) and in HCS (8.57%,3 out of 35) (cut-off value 0.36; AUC = 0.89; *p*  <  0.0001, Figure 2B). Anti-HERV-Wenv _129–143_ Abs were detected in T1D patients (44.44%, 20 out of 45) and in HCs (8.89%, 4 out of 45) (cut-off value of 0.3630; AUC = 0.68; *p*  =  0.0019, Figure 2C); in patients with onset of T1D (69.23%, 18 out of 26) and in HCS (5.71%, 2 out of 35) (cut-off value 0.36; AUC = 0.86; *p*  <  0.0001, Figure 2D). 

Anti-HERV-Wenv _248–262_ Abs were detected in T1D patients (44.44%, 20 out of 45) and in HCs (6.67%, 3 out of 45) (cut-off value of 0.24; AUC = 0.73; *p*  <  0.0001, Figure 2E); in patients with onset of T1D 88.46%, 23 out of 26) and in HCS (8.57%, 3 out of 35) (cut-off value 0.24; AUC = 0.95; *p*  <  0.0001, Figure 2F). 

### 3.3. Analysis of Correlation between Anti-MAP and Anti-HERVenv Antibody Titers

**Figure 3:**
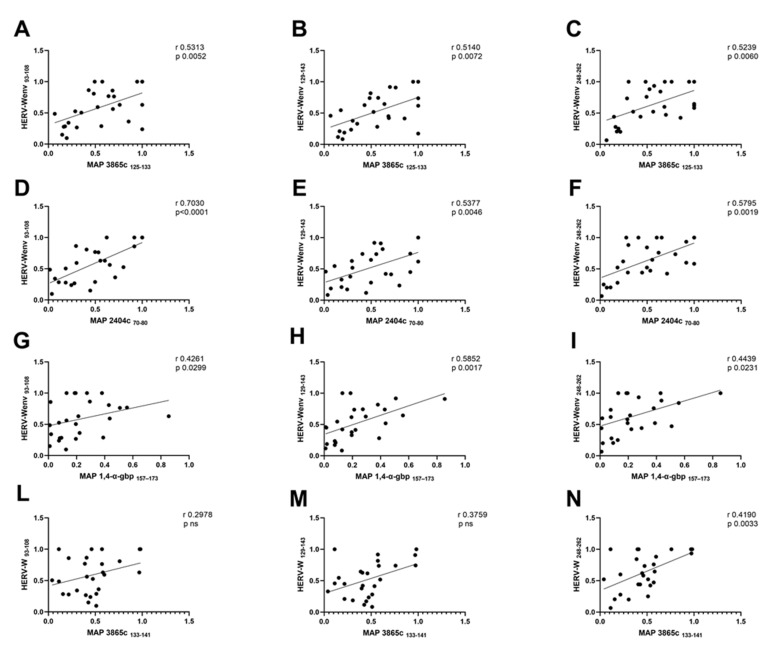
Scatter plot expressing correlation between MAP and HERV-Wenv derived peptides in a pediatric population with T1D at onset. The graphs show the correlation among MAP 3865c _125–133_ and HERV-Wenv _93–108_ (**A**); HERV-Wenv _129–143_ (**B**); HERV-Wenv _248–262_ (**C**); Correlation among MAP 2404c _70–80_ and HERV-Wenv _93–108_ (**D**); HERV-Wenv _129–143_ (**E**); HERV-Wenv _248–262_ (**F**); Correlations among MAP 1,4-α-gbp _157–173_ with HERV-Wenv _93–108_ (**G**); with HERV-Wenv _129–143_ (**H**); HERV-Wenv _248–262_ (**I**). Finally, correlation among MAP 3865c _133–141_ with HERV-Wenv _93–108_ is shown in (**L**); HERV-Wenv _129–143_ (**M**); HERV-Wenv _248–262_ (**N**) in T1D onset patients.

The correlation analyses in Table 2 showed a positive correlation between the antibody response against MAP epitopes and HERV-W peptides in the patients with onset of T1D. Moderate correlations on the basis of their r value are explained below: high correlation (0.7 to 0.89), moderate correlation (0.5 to 0.69), low correlation (0.3 to 0.49), little if any correlation (0.00 to 0.29) [26]. A moderate correlation was found between MAP 3865c _125–133_ with HERV-Wenv _93–108_ (r = 0.53, *p* = 0.005, Figure 3A), between MAP 3865c _125–133_ and HERV-Wenv _129–143_ (r = 0.51, *p* = 0.007, Figure 3B), and between MAP 3865c _125–133_ and HERV-Wenv _248–262_ (r = 0.52, *p* = 0.006, Figure 3C).

A high correlation was found between MAP 2404c _70–80_ and HERV-Wenv _93–108_ (r = 0.70, *p* < 0.0001, Figure 3D), moderate correlations between MAP 2404c _70–80_ and HERV-Wenv _129–143_ (r = 0.54, *p* = 0.005, Figure 3E), and between MAP 2404c _70–80_ and HERV-Wenv _248–262_ (r = 0.58, *p* = 0.02, Figure 3F).

A low correlation was observed between MAP 1,4-α-gbp _157–173_ and HERV-Wenv _93–108_ (r = 0.43, *p* = 0.03, Figure 3G), and between MAP 1,4-α-gbp _157–173_ and HERV-Wenv _248–262_ (r = 0.44, *p* = 0.002, Figure 3I), while a moderate correlation between MAP 2404c _70–80_ and HERV-Wenv _129–143_ (r = 0.58, *p* = 0.002, Figure 3H).

Finally, a low correlation was found between MAP 3865c _133–141_ and HERV-Wenv _248–262_ (r = 0.23, *p* = 0.03, Figure 3N), whereas no significant correlation was found between MAP 3865c _133–141_ and HERV-Wenv _93–108_ as well as between MAP 3865c _133–141_ and HERV-Wenv _129–143_ (Figure 3L,M). 

## 4. Discussion

The incidence of type 1 diabetes in children has increased worldwide, and it is therefore important to find markers to enable early diagnosis and new treatments. Despite intensive research, the etiology of T1D remains unknown, and a multiplicity of etiological agents has been proposed as potential candidates to triggers autoimmunity in susceptible individuals with T1D [27].

The present study analyzed the potential involvement of MAP and HER-W as contributor factors in T1D etiopathogenesis, based on previous reports. Indeed, in recent studies a direct correlation between anti-MAP Abs and anti-HERV-Wenv has been observed in a cohort of patient with T1D [28].

This study demonstrates for the first time a high seroprevalence of anti-Map and anti-HERV-W Abs in pediatric patients with T1D from Sardinia. Our results support the hypothesis that both pathogens are linked to T1D etiology and also that HERV-W activation can be induced by certain infectious agent such as MAP. To note, a possible connection between MAP and HERV-W was also showed in multiple sclerosis (MS) where it was detected a partial decrease of anti-MAP Abs and anti-HERV-Wenv in the serum of patients following treatment with natalizumab [29].

These differences may be the first step in suggesting MAP infection as a plausible circumstance capable of inducing the expression of HERV-W antigens which in turn lead to immune imbalance. 

The significant results we have obtained are in line with this hypothesis, given the high reactivity expressed in patients at the onset for all peptide fragments evaluated of MAP and HERV-Wenv compared to a healthy population of corresponding age and geographical background but also compared to the population with diabetes developed for years. Interestingly, in young people receiving insulin therapy, we observed a lower antibody titer for HERV-Wenv than young people of the same age at the start of the disease. It was seen that in MAP infection Th17-derived cytokine genes were down-regulated, these play an important role in the early phase of mycobacterial infection [30], in particular IL-26, which induces immune cell initiation and direct pathogen killing [31] and IL-17F, which provides protective immunity against intracellular pathogens through modulation of the Th1 response and neutrophil recruitment [32,33]. 

MAP also influences the expression of interferon regulatory genes, with reduced expression of IRF4 inducing an under-regulation of the Th1 immune response, thus enhancing the persistent survival of MAP [34], and an over-regulation of IRF5 and IRF7 that may result in inhibition of T-cell proliferation through tryptophan depletion mediated by indoleamine 2,3-dioxygenase that subsequently leads to an immunosuppressive state [35].

Several factors may contribute to the transcriptional reactivation of HERV-W and may promote pathological conditions [36], some environmental stimuli such as infection immune factors and oxidative stress seems to contribute by hindering the binding of methyl groups transferred by DNA methyltransferases and enhancing gene expression following inhibition of histone deacetylases through interaction with specific binding sites in HERV promoter regions [37].

Adhesion of MAP to the mucosa lining the small intestine and subsequent uptake by M-cells and enterocytes may play a role in triggering antibody production [16] another contribution of MAP in inducing latent infection in humans could result from molecular mimicry with homologous epitopes such as ZnT8 and PI, leading to autoimmune responses [38,39]. 

In addition, oxidative processes have been demonstrated in patients with Crohn’s disease and in MAP-infected cattle, where an increase in selenium-dependent glutathione peroxidase (GPx) activity has been seen [40]. This probably contribute to HERV transcriptional reactivation by hindering the binding of methyl groups transferred by DNA methyltransferases and enhancing gene expression following inhibition of histone deacetylases and consequently promote disease conditions [37].

The hypothesis that MAP may be involved in HERV-W transactivation is also supported by the presence of highly expressed CD68+ in granulomatous lesions of infected goats, indicating high lysosome counts and acid phosphatase activity [41], similarly, increased expression of CD68+ infiltrating macrophages has been reported in the pancreas of T1D patients, in this case linked to HERV-W expression [22]. 

As humans are not the main target of MAP, it may act indirectly on immune homeostasis as a consequence of survival mechanisms that elude host defense against pathogens and conditions that favor the expression of HERV-W antigens. In conclusion, we have demonstrated high seropositivity against MAP and HERV-W peptides with a significant prevalence at disease onset and at the same time the moderate and high correlations present between the two agents; further investigation would help to elucidate the role of MAP as a plausible trigger for the expression of HERV-W antigens which in turn lead to immune imbalance and subsequent disease onset. Additional follow up studies of Ab-titers over longer time frames are necessary to determine the role of both pathogens in T1D.

## Figures and Tables

**Table 1:** Epitopes identified in MAP and HERV-Wenv.

Peptides	Position	Sequence
MAP 3865c	aa 125–133	MIAVALAGL
MAP 3865c	aa 133–141	LAANFVVAL
MAP 2404c	aa 70–80	RGFVVLPVTRRDVTDV
MAP 1,4-α-gbp	aa 157–173	GTVELLGGPLAHPFQPL
HERV-Wenv	aa 93–108	NPSCPGGLGVTVCWTY
HERV-Wenv	aa 129–143	VKEVISQLTRVRHGT
HERV-Wenv	aa 248–262	NSQCIRWVTPPTQIV

aa: Aminoacids.

**Table 2:** Relationship between HERV-Wenv antigens and MAP-derived epitopes expressed as r. Values were obtained based on all available samples for single populations. Values were obtained based on all samples at the onset of T1D.

MAP Antigen	HERV-Wenv 93–108	HERV-Wenv 129–143	HERV-Wenv 248–262
MAP 3865c _125–133_	r = 0.5313 *p* = 0.0052	r = 0.5140 *p* = 0.0072	r = 0.5239 *p* = 0.0060
MAP 2404c _70–80_	r = 0.7030 *p* < 0.0001	r = 0.5377 *p* = 0.0046	r = 0.5795 *p* = 0.0019
MAP 1,4-α-gbp _157–173_	r = 0.4261 *p* = 0.0299	r = 0.5852 *p* = 0.0017	r = 0.4439 *p* = 0.0213
MAP 3865c _133–141_	r = 0.2978 *p* ns	r = 0.3759 *p* ns	r = 0.4190 *p* = 0.0311

## Supplementary Materials

The following are available online at https://www.mdpi.com/article/10.3390/pathogens10091135/s1, Table S1: Clinical Data T1D (*n* = 71).
