# Supplementary material for: HERV-W and Mycobacterium avium subspecies paratuberculosis Are at Play in Pediatric Patients at Onset of Type 1 Diabetes
Source: Pathogens. 2021 Sep 3;10(9):1135. doi: 10.3390/pathogens10091135 (PMC8471288; doi:10.3390/pathogens10091135)
Supplement: Supplementary file 1 [file pathogens-10-01135-s001.zip › pathogens-1328506-supplementary.pdf]

Table S1. Clinical Data T1D (*n* = 71).

ID	Disease T1D	Comorbidity	Familiarity Disease	HbA1c
T1D 1	Onset	--	Grandmother with T1D	13.5%
T1D 2	T1D for 5 years	Hashimoto thyroiditis (HT)	Two aunts with T1D	--
T1D 4	Onset	--	Grandmother with T1D / Paternal uncle with T1D	10.1%
T1D 5	T1D for 4 years	Coeliac	Uncle with T1D	--
T1D 6	T1D for 4 years	--	Mother with HT	--
T1D 7	T1D for 11 years	--	Grandmother with T1D	--
T1D 8	T1D for 12 years	--	Uncle with T1D	--
T1D 9	Onset	--	Grandfather with T1D	11.3%
T1D 10	Onset	--	Grandmother with T1D / cousin with T1D	10.1%
T1D 11	T1D for 8 years	--	--	--
T1D 12	T1D for 4 years	--	--	--
T1D 13	T1D for 6 years	--	Mother with T1D	--
T1D 14	T1D for 6 years	--	Cousin with T1D	--
T1D 15	T1D for 4 years	--	--	--
T1D 16	T1D for 8 years	--	--	--
T1D 17	Onset	--	--	--
T1D 18	Onset	HT	Grandmother with T2D/ Maternal uncle with T1D	10.9%
T1D 19	T1D for 10 years	Coeliac / HT	Brother with T1D / cousin with T1D	__
T1D 21	T1D for 10 years	--	--	--
T1D 22	T1D for 4 years	--	Grandfather with T2D	--
T1D 23	Onset	HT	--	10.2%
T1D 24	T1D for 4 years	--	Grandfather with T2D	--
T1D 25	Onset	--	--	13.2%
T1D 26	Onset	Coeliac / HT	--	--
T1D 27	T1D for 1 years	HT	--	--
T1D 28	Onset	--	--	13.2%
T1D 30	T1D for 6 years	--	--	--
T1D 31	Onset	--	Mother with T1D / Father with T2D / cousin with T1D	13.5%
T1D 33	T1D for 9 years	--	--	--
T1D 35	T1D for 7 years	--	--	--
T1D 36	T1D for 10 years	--	--	--
T1D 37	Onset	HT	--	10.4%
T1D 38	T1D for 1 years	--	Brother with T1D / Father with T2D	--
T1D 39	Onset	--	Grandmother with T2D	10.7%
T1D 40	T1D for 10 years	--	Uncle with T1D / grandmother with T2D	--
T1D 41	T1D for 5 years	--	Father with T1D	--
T1D 43	T1D for 3 years	--	Father with T1D	--
T1D 44	Onset	--	--	11%
T1D 46	Onset	--	Cousin with T1D	9.5%
T1D 47	Onset	--	--	11.6%
T1D 48	T1D for 4 years	--	Aunt with T1D	
T1D 49	Onset	--	--	10.2%
T1D 50	Onset	--	--	10.4%
T1D 51	Onset	--	Three cousins with T1D / Grandmother with T2D	13%
T1D 52	T1D for 2 years	--	--	--
T1D 53	T1D for 5 years	--	--	--
T1D 54	T1D for 3 years	HT	--	--
T1D 55	T1D for 10 years	--	--	--
T1D 56	Onset	--	--	10.2%
T1D 57	Onset	--	Cousin with T1D / aunt with T1D	9.8%
T1D 58	Onset	--	Grandmother with T2D / cousin with T1D	11.5%
T1D 59	T1D for 4 years	HT	Brother with T1D /	--
T1D 60	T1D for 14 years	--	Uncle with T1D	--

T1D 61	T1D for 12 years	--	--	--
T1D 62	T1D for 4 years	--	--	--
T1D 63	T1D for 9 years	--	--	--
T1D 64	T1D for 5 years	--	--	--
T1D 65	T1D for 6 years	--	--	--
T1D 66	T1D for 5 years	Coeliac / HT	--	--
T1D 67	T1D for 1 years	--	--	--
T1D 68	Onset	--	--	11%
T1D 69	T1D for 10 years	--	--	--
T1D 70	T1D for 8 years	--	Uncle with T1D	--
T1D 71	T1D for 7 years	--	Grandfather with T2D	--
T1D 72	T1D for 8 years	--	--	--
T1D 73	T1D for 6 years	--	--	--
T1D 74	Onset	--	Father's uncle with T1D	10.4%
T1D 75	Onset	--	Grandmother with T1D / grandfather with T2D	--
T1D 76	Onset	--	Cousin with T1D	12.3%
T1D 77	T1D for 2 years	--	Sister with T1D	--
T1D 78	T1D for 12 years	--	--	--
